# Preference and usage pattern of mobile medical apps for drug information purposes among hospital pharmacists in Sarawak, Malaysia

**DOI:** 10.1186/s12911-022-01949-9

**Published:** 2022-07-29

**Authors:** Boon Phiaw Kho, Sheng Ming Andy Wong, Jin Wei Timothy Chiu, Eon Liew

**Affiliations:** Department of Pharmacy, Sarawak General Hospital, Ministry of Health Malaysia, 93586 Tun Ahmad Zaidi Adruce Road, Kuching, Sarawak Malaysia

**Keywords:** Pharmacy service, hospital, Mobile applications, Drug information services, Decision support systems, clinical

## Abstract

**Introduction:**

Pharmacists are frequent users of mobile medical apps (MMA) for drug information (DI) and clinical decision-making purposes. However, the wide range of available MMA may be of variable credibility and results in heterogeneous recommendations. The need for subscription may also influence choice of apps.

**Objective:**

The objective of this study was to determine the usage pattern of MMA among hospital pharmacists, including their perceptions and factors affecting their choice of apps.

**Methods:**

This cross-sectional study required respondents to fill in an online questionnaire. The questionnaire included sections on respondents' demographic data, MMA usage pattern, perceived usefulness and opinion on subscription fees. Items were adapted from available literature and validated locally. It was made accessible for 6 weeks starting November 2019 for all pharmacists working in the 23 public hospitals in Sarawak to response (universal sampling). Collected data were analysed using descriptive and inferential statistics.

**Results:**

A response rate of 37.2% was achieved (n = 162). Respondents were heavily reliant on MMA, with 78.4% accessing them multiple times daily. The majority also agreed that MMA contain correct and up-to-date information. A median of 5 apps were downloaded, suggesting an ultimate app catering for all DI needs was lacking. The Malaysian Drug Formulary was the most downloaded app (88.3%), whereas Lexicomp**®** was the most “well-rounded” in terms of functionality. Clinical pharmacists were significantly more likely to purchase MMA, in particular UpToDate**®** (*p* < 0.01) due to their need to access clinical updates. Respondents highly recommended institutional access for either UpToDate**®** or Lexicomp**®** be made available. Pre-registration pharmacists should be guided on judicious MMA usage, as they downloaded significantly more apps and were more likely to indicate not knowing which DI recommendation to follow (both *p* < 0.01).

**Conclusion:**

MMA has become an indispensable tool for hospital pharmacists, however there was a tendency to download multiple apps for DI needs. Institutional access can be considered for credible apps identified to ensure accuracy and uniformity of DI recommendations, with purchase decision made after surveying the needs and preferences of end users.

**Supplementary Information:**

The online version contains supplementary material available at 10.1186/s12911-022-01949-9.

## Introduction

The availability of mobile applications or commonly known as mobile apps had made smartphones an ubiquitous and indispensable gadget for healthcare professionals in their daily work nowadays [1]. Likewise, pharmacists had been using mobile medical apps (MMA) in their daily practice, primarily as drug information and decision making tools [2, 3]. Usage of MMA purportedly enhanced pharmacists' productivity, with the availability of information at their fingertips enabling efficient delivery of patient care [2]. Indeed, MMA are developed with the objective of improving efficacy in sound clinical decision-making with lower error rate, the quality of data management as well as access to better healthcare [4]. The development of MMA is timely to cater for the increasing need for drug information demanded by the expanding complexity and scope of pharmacy services [5].

Some of the commonly used MMA among healthcare professionals, especially in the context of pharmacists, are drug references, clinical practice guidelines, medical calculators and apps for work productivity. Drug references apps are often used to locate various information of medications, from indication, dosage to side effects [1]. Examples of well-established drug references mobile apps are Micromedex**®**, Lexicomp**®**, Medscape**®** and Epocrates**®**. Clinical practice references apps meanwhile are decision making tools used to recommend evidence based solutions at point-of-care, with popular apps include Sanford Guide to Antimicrobial Therapy**®** and UpToDate**®**. [2] There are also a number of apps for work productivity such as Evernote**®**, Wunderlist**®**, Dropbox**®** and Google Drive, as well as medical calculators such as MedCalc Pro**®** and Calculate by QxMD**® **[2]. Among Malaysian pharmacists, drug information for dosage recommendation, adverse drug reaction, drug-drug interaction and also dosage recommendation for special population were often sourced using smartphones or tablets [3].

Despite their ubiquity, numerous concerns regarding the usage and indeed increasing reliance of pharmacists on MMA exist. The lack of evidence and professional medical involvement in the design and development of some MMA raised concerns regarding the reliability and accuracy of their medical content, and the potential adverse consequences to patient safety [6]. This complexity was heighten by the difficulties faced in adjudicating the accuracy and reliability of available MMA, as they are all created by independent developers without oversight by any regulatory bodies [7]. There is now a mobile health apps overload, increasing the difficulty in finding useful and applicable apps [4]. The wide usage of MMA also resulted in a variety of answers for a question, depending on the source of information. This is in contrast with traditional drug information, which was provided based on a few notable established hardcopy tertiary sources thus ensuring uniformity in recommendations. Besides, apps are also too fragmented, with most apps only good for performing certain functions rather than being a comprehensive, multipurpose suite. As more established MMA are subjected to subscription charges, this may also increase tendency for pharmacists to opt for free apps that may be less reliable, compromising the quality of drug information provided [8]. Indeed, choice of MMA among pharmacy students were found to be influenced by the availability of institutional subscriptions [9, 10].

Pharmacists in Malaysia predominantly used MMA to search for drug information, especially dosage recommendations [3]. Average usage was once per day, with Medscape® and Micromedex® being the most downloaded apps [3]. Pharmacy students in Canada were found to use between 1 and 3 MMA for drug information purposes, with nearly half utilizing them more than once per day [9]. Various factors were found to influence the usage of MMA among health professionals. Well-established factors include perceived ease of use, perceived usefulness and peer influence [11, 12]. For drug information purposes, availability of dosing and drug interaction information, as well as ease of use were important considerations [13]. To the best of our knowledge, perceptions of working pharmacists on paying for MMA for drug information purposes had yet to be explored. Opinions regarding paid subscriptions were gathered among pharmacy students, with paid MMA perceived to be superior in terms of accuracy, comprehensiveness, and currency of information by pharmacy students [9]. They were also more likely to use free apps compared to those requiring paid subscriptions [10].

This study aimed to determine the current pattern of MMA usage for drug information and clinical decision making purposes among pharmacists working in public hospitals in the state of Sarawak, Malaysia, including the number and types of apps used, their functions and frequency of usage. Their perceptions on the usefulness of MMA, the need for paid subscriptions and barriers faced in using them will also be explored.

## Methods

### Study design and setting

This cross-sectional survey was conducted online using the Google Forms (Google Inc., Mountain View, CA) web application. It was carried out among registered pharmacists working in all 23 public hospitals in the state of Sarawak, Malaysia. Pharmacists working in public health clinics or private institutions were not included.

### Recruitment and sampling

Universal sampling method was used in this study, where all pharmacists, either fully registered (FRP) or provisionally registered (PRP) working in the targeted hospitals were recruited. A sampling frame was available, as the Pharmacy Services Division, Sarawak State Health Department was able to provide an up-to-date listing of total number of pharmacists in each facility. At the time of data collection, there were 435 eligible participants. Based on a margin of error of 7.5%, confidence interval of 95% and response distribution of 50%, the minimum sample size required as calculated using the Raosoft website (http://www.raosoft.com/samplesize.html) was 123. Universal sampling was chosen as the response rate of pharmacists was predicted to be between 25 and 40%, based on previous studies utilising similar distribution methods on similar population [14, 15]. Response distribution of 50% was chosen as it will yield the most conservative minimum sample size required, as data on proportion of respondents downloading each app were not available.

### Development and validation of survey instrument

The questionnaire comprised 5 sections: social demographic data (6 items), pattern of MMA usage (list of MMA to choose apps downloaded, functions of each app and 1 item on the reliance on the app), perceptions on the need for subscription for MMA (5 items using 5 point Likert-like format, and 2 items on current apps purchased), general perceptions on the usefulness and trustworthiness of MMA (5 items using 5 point Likert-like format) and barriers faced in using MMA (a drop down list with 6 options to select). The list of commonly used apps was based on current apps downloaded by a convenient sample of 10 pharmacists working in SGH and the list from previous studies [2, 3, 16]. Items in other sections were self-constructed based on the literature review conducted [1, 11, 17].

Face and content validation of the questionnaire was done by having 3 senior pharmacists working in Sarawak General Hospital going through the items to determine their appropriateness in terms of wording, language use, and intention of the survey. Amendments were made based on comments by the pharmacists. It was then piloted among 10 pharmacists who were recently transferred from SGH to health clinics. All these pharmacists were not included in the actual data collection.

### Data collection and analysis

The link to assess the survey form was disseminated via email and WhatsApp messaging service (WhatsApp LLC, Menlo Park, CA) to participants via the chief pharmacist of the hospitals involved in late November 2019. A reminder email was sent 4 weeks later. The link was active for a total of 6 weeks. Respondents were classified as early responders if they responded within the first 4 weeks, and late responders if they responded in the last 2 weeks.

The collected data were extracted from Google Forms web application to be entered and recoded into SPSS software version 20 (IBM Corporation, Armonk, NY). Associations between social demographic profile of respondents and their pattern of MMA usage, perceptions on need of subscription, usefulness and trustworthiness of MMA as well as barriers faced were identified using Chi-square test and Pearson correlation coefficient. Statistical significance was set at a *p*-value of < 0.05.

## Results

### Demographic profile of respondents

Out of a total of 435 potential respondents, 162 responses were received (response rate: 37.2%). Demographic profile and the work position of respondents are listed in Table 1. Comparison of the demographic characteristics of early and late responders found no significant differences, indicating that non-responders were unlikely to have significant impact on study findings [18].

**Table 1:** Social demographic data and general usage pattern of mobile medical apps among respondents

Characteristics	Frequency (n)	Percentage (%)
*Gender*		
Male	29	17.9%
Female	133	82.1%
*Age*		
23–30 years old	110	67.9%
31–40 years old	52	32.1%
*Highest level of education*		
Bachelor degree	140	86.4%
Clinical specialisation	13	8.0%
Masters degree	9	5.6%
*Designation*		
Fully registered pharmacist	116	71.6%
Provisionally registered pharmacist (interns)	46	28.4%
*Type of hospital*		
Major/state hospital	103	63.6%
Minor & non-specialist hospital	59	36.4%
*Department*		
Clinical	31	19.1%
Outpatient and inpatient services	52	32.1%
Drug information	7	04.3%
Others	26	16.1%
Interns (rotation to multiple departments)	46	28.4%
*MMA downloaded for drug information purposes (only apps* > *50% usage shown)*		
My BlueBook/DIY formulary**®** (Malaysian Drug Formulary)	152	93.8%
Medscape**®**	130	80.2%
Micromedex**®**	113	69.8%
Lexicomp**®**	112	69.1%
MIMS Malaysia/MIMS gateway**®**	94	58.0%
UpToDate**®**	86	53.1%
*MMA downloaded for other clinical purposes*		
eGFR calculations	69	42.6%
Other medication calculations	57	35.6%
Renal dose adjustment	116	72.0%
Drug counselling	78	48.4%
Treatment guidelines	93	58.5%
*Number of mobile medical apps downloaded*		
3 or less	40	24.7%
4 to 5	71	43.8%
6 or more	51	31.5%
*Frequency of use*		
More than once per day	127	78.4
Once or less per day	35	21.6

### Usage pattern of mobile medical apps

Results indicated that 78.4% of hospital pharmacists used MMA more than once per day. Three quarter of them had at least 4 MMA on their phones. Provisionally registered pharmacists (PRP) were more likely to use MMA more than once per day (x^2^ statistic = 4.37, df = 1, *p* = 0.037) and downloaded more MMA (x^2^ statistic = 11.22, df = 2, *p* = 0.004) when compared to their fully registered counterparts (FRP).

For drug information purposes, there were 6 MMA used by nearly half of the respondents, with the functions of the MMA being used characterised in Table 2. The Malaysian National Drug Formulary/My Blue Book**®** (M House Technology, Malaysia) was the most downloaded app, and mainly used for drug indication and drug dosage recommendation. Lexicomp**®** (Wolters Kluwer, Hudson, OH) seemed to be the most comprehensive app in terms of drug information functionality for respondents, as it recorded the highest proportion of usage among the available apps for drug dosage, adverse drug interaction and drug interaction. On the other hand, UpToDate**®** (Wolters Kluwer, Hudson, OH), was the app with the highest proportion of usage for clinical updates. Various MMA were also downloaded to facilitate clinical functions other than drug information, especially renal dose adjustment calculations and treatment guidelines, as indicated in Table 1.

**Table 2:** Type and drug information functions of MMA downloaded by hospital pharmacists

MMA downloaded	No. of users, n (%)	What drug information functions were utilised				
		Drug indication	Drug dosage	Adverse drug reaction	Drug interaction	Clinical updates
My Blue Book**®**	152	**131 (91.6)**	115 (80.4)	22 (15.4)	10 (7.0)	7 (4.9)
Medscape**®**	130	76 (64.4)	79 (66.9)	44 (37.3)	44 (37.3)	48 (40.7)
Micromedex**®**	113	72 (70.6)	87 (85.3)	53 (52.0)	46 (45.1)	17 (16.7)
Lexicomp**®**	112	96 (85.0)	**107 (94.7)**	**73 (64.6)**	**65 (57.5)**	38 (33.6)
MIMS**®**	94	50 (64.1)	58 (74.4)	20 (25.6)	14 (17.9)	17 (21.8)
UpToDate**®**	86	64 (69.6)	72 (78.3)	43 (46.7)	34 (37.0)	**68 (73.9)**

### Opinion on the need of paid subscription for MMA

As indicated in Table 3, the majority of respondents advocated for free access to the MMA they are using, or for the government to purchase institutional access. Respondents were divided on whether free MMA are sufficient to cater for their drug information needs. Most did not mind using alternative means to access paid subscription. Sixty-six respondents (40.7%) had MMA with paid subscription, mainly UpToDate**®** (53 respondents). Among FRPs, those working in sections recognised as heavy drug information users, namely clinical and drug information pharmacists were significantly more likely to purchase MMA (x^2^ statistic = 18.02, df = 2, *p* < 0.001), in particular UpToDate**®** (x^2^ statistic = 26.92, df = 2, *p* < 0.001).

**Table 3:** Level of agreement on statements regarding payment for MMA

Statement	Strongly disagree	Disagree	Neutral	Agree	Strongly agree
All mobile medical apps should be free of charge	3 (1.9)	7 (4.3)	28 (17.3)	32 (19.8)	92 (56.8)
Free mobile medical apps are sufficient to cater for my needs	17 (10.5)	29 (17.9)	47 (29.0)	38 (23.5)	31 (19.1)
The subscription fee charged by mobile medial apps are too costly	0 (0.0)	8 (4.9)	17 (10.5)	46 (28.4)	91 (56.2)
I do not mind using alternative means to access paid subscription (eg. free trial use, others' institutional access)	6 (3.7)	9 (5.6)	18 (11.1)	50 (30.9)	79 (48.8)
It is the obligation of the government to purchase MMA for our use, as we are using it for work purposes	0 (0.0)	1 (0.6)	8 (4.9)	41 (25.3)	112 (69.1)

Respondents were also asked to recommend MMA to be considered for institutional license. UpToDate**®** (86 respondents) together with Lexicomp**®** (81 respondents) were the runaway favourites. Clinical and drug information pharmacists were significantly more likely to recommend UpToDate**®** (x^2^ statistic = 9.19, df = 2, *p* = 0.010), whereas others favoured Lexicomp**®** (x^2^ statistic = 7.31, df = 2, *p* = 0.026).

### Perceived usefulness and trustworthiness of mobile medical apps

Nearly all respondents perceived information contained in MMA as correct and up-to-date, rendering them a useful and trustworthy companion for daily decision making and solving drug related enquiries (Table 4). The availability of multiple resources however resulted in a choice quandary, as heterogeneous recommendations by different apps made it difficult to determine which one to follow. PRPs are significantly more likely to agree with this statement compared to FRPs (t statistics, mean difference 0.52, 95% CI 0.20–0.84, *p* = 0.002). No significant differences were detected for other demographic variables.

**Table 4:** Perceived usefulness and trustworthiness of mobile medical apps utilised

Statement	Strongly disagree	Disagree	Neutral	Agree	Strongly agree
MMA help in daily decision making for drug choices and doses	0 (0.0)	2 (1.2)	3 (1.9)	45 (27.8)	112 (69.1)
MMA enable me to answer queries faster compared to conventional sources	0 (0.0)	1 (0.6)	4 (2.5)	37 (22.8)	120 (74.1)
I am confident that information contained in MMA are correct	0 (0.0)	2 (1.2)	13 (8.0)	72 (44.4)	75 (46.3)
I am confident information contained in MMA are up-to-date	0 (0.0)	2 (1.2)	19 (11.7)	75 (46.3)	66 (40.7)
I have problems deciding which recommendations to follow as they are not the same	3 (1.9)	28 (17.3)	56 (34.6)	57 (35.2)	18 (11.1)

### Barriers faced in using mobile medical apps

Lack of fast and stable internet was the predominant barrier faced by pharmacists in using MMA (78.4%). This may be due to hospital buildings having structures that obstruct Internet reception. Other barriers include not knowing which resources are available (25.9%), apps having complicated installation process (17.3%), not understanding how to use the resource (10.5%), not having the technology to use MMA (8.6%) and apps having interface that is not user-friendly (6.8%).

## Discussion

The findings that majority of hospital pharmacists who responded to the survey were reliant on MMA to perform their daily task, especially to access drug information and assist clinical decision making highlight the integral role of MMA for pharmacists nowadays. The frequency of access was higher compared to a similar study conducted in Malaysia in 2013, suggesting an increased reliance on MMA, to the point that it can be considered an indispensable tool [3]. Respondents also have high trust on the accuracy of the information, despite the various concerns regarding credibility raised in the literature [6, 7, 17]. This may be because that most of the MMA they were using were established apps being universally used by most pharmacists and pharmacy students [3, 9, 10]. Nevertheless, better regulation on MMA, including mandating external peer review and periodic assessment should be pursued to safeguard the integrity of the information, increasing the confidence of healthcare professionals to use them [6, 19].

The national formulary was the most downloaded app, demonstrating the importance of having a localized formulary app to facilitate drug information decision making. Having information on drugs available in their country or facility of practice, as well as restrictions of use will be a beneficial quick reference guide for hospital pharmacists. The fact that pharmacists downloaded multiple drug information apps, as well as apps that cater for specific clinical purposes seemed to indicate the lack of a single app that could cater for all their needs. The usage pattern of respondents suggested that they might have preference in using specific app to perform particular functions. This is consistent with a feature assessment among selected MMA conducted by Apidi et al. (2017), who concluded that drug information apps had largely similar but different sets of functionalities. Another review found that numerous drug information apps lacked details on drug interaction, dose adjustment and use in pregnancy or breastfeeding [20]. Thus, the creation of an ultimate MMA tailored to both the drug information and clinical needs of pharmacists may be an effort that is worthwhile to be pursued. However, the usage of several apps enables comparison and triangulation of recommendations provided. Respondents may had downloaded multiple apps to compare the information contained within. This double-checking can ensure that the information sought and provided is accurate and up-to-date. It can also prevent a single company dominating the whole medical treatment landscape by influencing treatment choices information [6, 21].

There were more respondents using MMA requiring paid subscriptions than those declaring that they purchased subscriptions, suggesting that some were using alternative means to access MMA. This may be attributed to the belief that the government as the employer is obliged to pay for institutional subscription, as well as the high cost of subscription. The Malaysian government did subscribe Micromedex**®** app and online access of MIMS gateway**®** for healthcare workers. However, given a choice, Lexicomp**®** seemed likely to be the preferred option for respondents [9]. Rationale of this preference is unknown and can be further explored, as both apps have similar features. Previous research comparing pharmacists’ preference on these two apps have contrasting findings [10, 13]. In a previous research conducted in Malaysia, more respondents were using Micromedex**®** compared to Lexicomp**® **[3]. A nationwide survey on the preference of pharmacists can be considered before committing institutional subscription, with data on preference and cost allowing better decision making on which MMA to purchase [13]. The current subscription may also be insufficient to cater for the needs of clinical pharmacists, who require MMA that focused on clinical or therapeutic management of diseases, rather than just drug information [11]. A study found that a significantly higher proportion of hospital pharmacists frequently utilised UptoDate® compared to their peers in community pharmacies [22]. Purchasing institutional access of UpToDate® specifically for them can be considered to better enhance patient care.

For pharmacy interns, the tendency to download more MMA and difficulties faced in deciding which recommendations to follow suggested a need for guidance by senior peers. The latter may assist by recommending appropriate MMA and providing insight on how to judge and decide which recommendations to follow shall different MMA provide diverging information. It is also important to improve their awareness on the various concerns surrounding the use of MMA [17]. Safe and judicious use of MMA is imperative to ensure the accuracy and credibility of drug information provided to other healthcare professionals and patients, as well as upholding patient confidentiality.

It was surprising that the lack of fast and stable Internet was the predominant barrier faced by respondents. At this age where high-speed Internet connectivity is expected, limited connection should no longer be a barrier to use MMA. Healthcare administrators should prioritise the availability of Internet connectivity and other facilitators of MMA adoption, including providing technical assistance, user-training as well as institutional subscriptions [21]. The lack of technical or technological barriers faced in using MMA can be attributed to the fact that the majority of pharmacists in Sarawak are relatively young, hence are more adept at using smartphones. Besides, the perceived usefulness and trustworthiness of MMA will likely overcome most resistance towards their use in daily practice [21].

## Limitations

Results were based on participants’ self-reported data, thus may be subjected to recall and social desirability bias. The response rate is lower than desired. One possible reason is that we were unable to exclude participants who did not use MMA due to the nature of our sampling and data collection methods, hence they contributed to the denominator. Pharmacists who did not use MMA were unlikely to response to the survey; only one participant indicated that he/she did not use MMA. Distribution of survey link was also dependent on the head of pharmacy departments of respective hospitals. It cannot be guaranteed that they did send the link to all eligible staff. Nonetheless, our response rate was higher compared to a similar study carried out in Canada, which recorded 16% complete response from pharmacists surveyed [13]. Options for functional usage of MMA were also not comprehensive, for example finding information on safety in pregnancy and lactation, IV compatibility or using the in-built dose calculator function available in some of the apps were not covered. Some respondents might had preferred using website-based applications on their smartphones rather than or in combination with MMA, which was not covered by this study. Despite being carried out in the biggest state in Malaysia covering 23 hospitals, the results may not be generalizable to pharmacists in other locations. Relationship between type of hospitals and place of work with MMA downloaded were not analyzed. Further study looking at this area is required as it is important to identify what type of MMA is suitable for which type of hospitals (which provide different specialization) as this will involve budget estimation in providing the MMA from the Malaysian Ministry of Health point of view.

## Conclusion

Mobile medical apps (MMA) have become an indispensable tool for hospital pharmacists. Most pharmacists however used multiple apps for drug information purposes, suggesting that there is yet to be a dominant or comprehensive app. Their utility and usage pattern are influenced by their functionality, perceived usefulness and trustworthiness as well as subscription cost. Institutional access can be considered for credible apps identified to ensure accuracy and uniformity of drug information recommendations, as well as to support the practice needs of clinical pharmacists. It is recommended for purchase decision to be taken after obtaining feedback from the end-users. This will ensure the purchase of apps that are appealing to both stakeholders, with the subsequent anticipated high usage providing value-for-money for the subscribing institution as well as enhancing patient care. A survey similar to this can be useful to elucidate their needs and preferences.

## Supplementary Information

**Additional file 1.**

## Data Availability

The data that support the findings of this study and the questionnaire used are available in the Additional file 1 attached.
